# Taxonomic novelties of two *Neosulcatispora* species (*Pleosporales*, *Phaeosphaeriaceae*) associated with *Agave vera-cruz* plants in a dry-hot valley of Yunnan, China

**DOI:** 10.3897/mycokeys.142.213343

**Published:** 2026-10-26

**Authors:** Lakmali S. Dissanayake, Rungtiwa Phookamsak, Sajeewa S. N. Maharachchikumbura, Ahmed E. Al-Sabri, Jianchu Xu, Dhanushka N. Wanasinghe

**Affiliations:** 1 Department of Economic Plants and Biotechnology, Yunnan Key Laboratory for Wild Plant Resources, Kunming Institute of Botany, Chinese Academy of Sciences, Kunming 650201, Yunnan, China Department of Economic Plants and Biotechnology, Yunnan Key Laboratory for Wild Plant Resources, Kunming Institute of Botany, Chinese Academy of Sciences Kunming China; 2 Centre for Mountain Futures, Kunming Institute of Botany, Chinese Academy of Sciences, Kunming, 650201, China Centre for Mountain Futures, Kunming Institute of Botany, Chinese Academy of Sciences Kunming China; 3 School of Life Science and Technology, Center for Informational Biology, University of Electronic Science and Technology of China, Chengdu 611731, China School of Life Science and Technology, Center for Informational Biology, University of Electronic Science and Technology of China Chengdu China; 4 Department of Botany and Microbiology, College of Science, King Saud University, P.O. Box 2455, Riyadh, 11451, Saudi Arabia Department of Botany and Microbiology, College of Science, King Saud University Riyadh Saudi Arabia

**Keywords:** *Asparagaceae* host, coelomycetous fungi, *
Dothideomycetes
*, Honghe dry-hot valleys, integrative taxonomy, southwestern China

## Abstract

*Neosulcatispora* is a poorly known coelomycetous genus of *Phaeosphaeriaceae*, previously represented by two species. During a survey of microfungi associated with dead leaves of *Agave vera-cruz* in the dry-hot valley of Honghe County, Yunnan Province, China, five isolates representing two unknown lineages were obtained. Morphology, culture characteristics and a multi-locus phylogenetic analysis of concatenated SSU, LSU, ITS, *tef1-α* and *rpb2* sequence data support their recognition as two new species viz. *Neosulcatispora agavicola***sp. nov**. and *N. hongheensis***sp. nov**. *Neosulcatispora agavicola* is characterized by relatively large, semi-immersed to erumpent conidiomata and golden-brown to dark brown, 1-septate, longitudinally striate conidia. *Neosulcatispora hongheensis* has smaller conidiomata and mature, 3–4-septate conidia. The new taxa form independent lineages within the clade containing the type species, *N. agaves*. In contrast, the ex-type strain of *N. strelitziae* is recovered elsewhere in *Phaeosphaeriaceae* in the present analysis, indicating that the generic placement of *N. strelitziae* requires reassessment using broader taxon and locus sampling. These findings increase the known diversity of *Neosulcatispora* sensu stricto and emphasize the dry-hot valleys of southwestern China as an understudied niche of undescribed plant-associated microfungi.

## Introduction

*Phaeosphaeriaceae* is a species-rich and ecologically diverse family within *Pleosporales*, comprising saprobic, endophytic, pathogenic, and lichenicolous fungi on a wide range of herbaceous and woody substrates (Lawrey et al. 2012; Phookamsak et al. 2014a; Wanasinghe and Maharachchikumbura 2023). Grasses and other monocotyledonous plants are particularly important hosts and continued sampling has revealed additional lineages and host associations within the family (Karunarathna et al. 2017; Tennakoon et al. 2020). Nevertheless, the taxonomic circumscription of *Phaeosphaeriaceae* and several of its genera have remained challenging. Overlapping and convergent morphological characters, particularly among coelomycetous asexual morphs, have obscured generic and species boundaries, while molecular evidence has shown that morphology-based classifications do not always reflect phylogenetic relationships. The comprehensive revision by Phookamsak et al. (2014a), followed by studies of ophiobolus-like lineages (Phookamsak et al. 2017), Asian karst collections (Zhang et al. 2019), and taxa from ecologically diverse habitats (Wanasinghe and Maharachchikumbura 2023), progressively refined the classification of the family and demonstrated the importance of integrating morphological and molecular evidence. Contemporary treatments of *Phaeosphaeriaceae* combine detailed micromorphology and ecological information with multilocus phylogenetic analyses (Wijayawardene et al. 2013; Wanasinghe and Maharachchikumbura 2023).

Recent surveys of herbaceous and woody substrates in Yunnan have also demonstrated that underexplored habitats continue to yield fungal taxonomic novelties (Gao et al. 2024; Wanasinghe et al. 2025). Recent studies across *Pleosporales* and other fungal groups illustrate the effectiveness of combining morphological evidence with multigene phylogenetic analyses for species delimitation and taxonomic placement (Htet et al. 2024; Hussain et al. 2024; Dela Cruz et al. 2025; Thambugala et al. 2025). These developments provide a clear rationale for applying an integrative morpho-molecular approach to the poorly sampled genus *Neosulcatispora*.

*Neosulcatispora* was established for *N. agaves*, a coelomycetous fungus described from leaves of *Agave vera-cruz* in La Réunion (Crous et al. 2015). The genus is characterized by pycnidial conidiomata, a wall composed of brown cells of *textura angularis*, phialidic conidiogenous cells and pigmented, longitudinally striate conidia. A second species, *N. strelitziae*, was subsequently introduced from leaf spots of *Strelitzia nicolai* in South Africa (Crous et al. 2016). However, the genus remains poorly sampled and its phylogenetic circumscription, host range, and morphological variation have not been evaluated using extensive multilocus phylogeny.

The dry-hot valleys of southwestern China experience high temperatures, pronounced seasonality, low precipitation during the dry season and strong solar radiation (Zhou et al. 2022; Sun et al. 2025). These conditions support drought-adapted vegetation and may select for distinctive fungal communities (Wu et al. 2018; Gao et al. 2025; Zhong et al. 2025). Recent surveys in Yunnan have revealed numerous taxonomic novelties from bamboo, herbaceous plants, woody litter, and cultivated or naturalized hosts (Dissanayake et al. 2024; Gao et al. 2024; Li et al. 2024; Luo et al. 2024). In particular, investigations of dead woody litter in the Honghe dry-hot valleys demonstrated that these habitats harbour substantial and still incompletely documented microfungal diversity (Wanasinghe et al. 2025).

*Agave vera-cruz* is a drought-tolerant monocotyledonous plant cultivated or naturalized in dry landscapes. Although the type species of *Neosulcatispora* was described from this host, fungal diversity associated with *Agave* in Asian dry-hot valleys has received little attention. During a survey of saprobic microfungi on dead *A. vera-cruz* leaves in Honghe County, we obtained five isolates with neosulcatispora-like asexual morphs. This study aims to (1) determine their phylogenetic placement using a five-locus dataset, (2) describe and illustrate the taxa based on morphology and culture characteristics and (3) reassess the current phylogenetic limits of *Neosulcatispora*.

## Materials and methods

### Sampling, specimen examination and isolation

Dead leaves of *Agave vera-cruz* were collected on 14 January 2026 from a dry-hot valley habitat in Honghe County, Honghe Hani and Yi Autonomous Prefecture, Yunnan Province, China (23.2452°N, 102.1546°E; 762 m a.s.l.). The collection site lies within the low-elevation Yuanjiang–Honghe dry-hot valley system, which is characterized by deeply incised valleys, steep slopes, strong solar radiation, high evaporative demand and pronounced seasonal water limitation (Wang et al. 2024). The region has a tropical semi-arid valley climate and January falls within the regional dry season, which generally extends from November to April. The natural vegetation of these valleys has a savanna-like appearance, comprising a predominantly herbaceous layer interspersed with drought-adapted shrubs and scattered trees (Zhu et al. 2024). The landscape is ecologically fragile and is frequently associated with sparse vegetation cover, eroded and nutrient-poor soils and localized land degradation caused by the interaction of steep terrain, concentrated seasonal rainfall and human disturbance (Wanasinghe et al. 2025; Wang et al. 2024).

Samples were placed separately in clean paper bags, labelled and transported to the laboratory for examination. The host material was examined under a stereomicroscope for the presence of fungal reproductive structures. Conidiomata visible on the leaf surface were photographed and carefully removed or sectioned for subsequent morphological examination and single-conidial isolation. Single-conidial isolation was performed by transferring individual conidia from sporulating conidiomata onto potato dextrose agar (PDA). Germinated conidia were examined under a Motic SMZ 168 Stereo Zoom microscope, and then transferred to potato dextrose agar [PDA; potato extract 4 g/L (equivalent to 200 g of infusion from potatoes), glucose 20 g/L, agar 15 g/L] for further analysis, such as DNA extraction, growth rate measurements and cultural characterization. Type specimens, including the holotypes were deposited in the Herbarium of Cryptogams, Kunming Institute of Botany, Chinese Academy of Sciences (**KUN-HKAS**), Kunming, China. The living cultures were deposited at the Kunming Institute of Botany Culture Collection (**KUNCC**), Kunming, China. The new taxa were registered in MycoBank (https:/www.mycobank.org).

### Morphological observations

The morphology of macro-/micro-structures was observed as described in Senanayake et al. (2020). Hand sections of the conidiomata were mounted in distilled water and the following characteristics were examined and measured: conidiomata diameter, height, color and shape; width of peridium, length and width (at the widest point) of conidia. Images were captured with a Nikon ECLIPSE Ni compound microscope fitted with a Canon EOS 600D camera. Culture characteristics were recorded from colonies grown on PDA at 25 °C in the dark. Colony diameter, surface and reverse colors, margin, elevation and aerial mycelium were assessed after 30 days. Macroscopic images of colonies were documented using an iPhone 17 (Apple Inc., Cupertino, CA, USA) with daylight. Measurements were made with the Tarosoft (R) Image Frame Work program, and images used for figures were processed with Adobe Photoshop CS5 Extended version 10.0 software (Adobe Systems, San José, CA, USA).

### DNA extraction, PCR amplification and sequencing

The extraction of genomic DNA was performed in accordance with the methods of Wanasinghe et al. (2025), using the Biospin Fungus Genomic DNA Extraction Kit-BSC14S1 (BioFlux, P.R. China) following the guidelines of the manufacturer. The genomic DNA of the new isolates was stored at 4 °C for the polymerase chain reaction (PCR) and duplicated at –20 °C for long-term storage. Primers ITS5/ITS4 (White et al. 1990), LR0R/LR5 (Vilgalys and Hester 1990), NS1/NS4 (White et al. 1990), EF1-983F/EF1-2218R (Liu et al. 1999; Rehner and Buckley 2005), and fRPB2-5f/fRPB2-7cR (Liu et al. 1999) were used to amplify the DNA sequences of the internal transcribed spacers (ITS), partial 28S large subunit rDNA (LSU), partial 18S small subunit rDNA (SSU), translation elongation factor 1-α (*tef*1), and RNA polymerase II second largest subunit (*rpb*2). The protocols used for the PCR amplification (SSU, LSU, ITS and *tef*1) are used as in Wanasinghe and Maharachchikumbura (2023). The PCR amplification condition for *rpb*2 was set as denaturation at 98 °C for 2 minutes, followed by 35 cycles of denaturation at 98 °C for 10 seconds, annealing at 52 °C for 10 seconds and extension at 72 °C for 20 seconds, with a final extension step at 72 °C for 5 minutes. The amplified PCR fragments were then sent to a private company for sequencing (Shenggong Biotechnology Co. Ltd., Shanghai, P.R. China). The nucleotide sequence data obtained were deposited in GenBank (Table 1).

**Table 1.** GenBank accession numbers of sequences used for the phylogenetic analyses.

**Species**	**Strain**	**GenBank Accession Numbers**					**References**
		**ITS**	**LSU**	**SSU**	***tef*1**	***rpb*2**	
*Acericola italica*	MFLUCC 13-0609 T	NR_156344	NG_057143	NG_063642	–	–	Hyde et al. (2017)
*Alloneottiosporina thailandica*	MFLUCC 15-0576 T	NR_169715	NG_073831	NG_070332	MT454002	MT432196	Li et al. (2020)
*Arezzomyces cytisi*	MFLUCC 15-0649 T	NR_153533	NG_057083	NG_063584	–	–	Marin-Felix et al. (2019)
*Banksiophoma australiensis*	CBS 142163 T	KY979739	KY979794	–	–	–	Crous et al. (2017)
*Bhagirathimyces himalayensis*	NFCCI 4580 T	NR_168816	NG_068644	NG_068414	MT091965	–	Hyde et al. (2020)
*Bhatiellae rosae*	MFLUCC 17-0664 T	NR_157505	NG_058718	NG_061286	–	–	Wanasinghe et al. (2018)
*Brunneomurispora lonicerae*	KUMCC 18-0157 T	NR_164299	NG_070462	NG_067697	MK359064	MK359079	Phookamsak et al. (2019)
*Camarosporioides phragmitis*	MFLUCC 13-0365 T	NR_153925	NG_058954	KX572350	KX572354	–	Hyde et al. (2016)
*Chaetosphaeronema clematidicola*	MFLUCC 17-2151 T	MT310619	MT214574	NG_070660	MT394633	MT394694	Phukhamsakda et al. (2020)
*Dematiopleospora salsolae*	MFLUCC 17-0828 T	NR_157514	NG_059184	NG_063679	MG829201	MG829254	Wanasinghe et al. (2018)
*Didymocyrtis consimilis*	CBS 129141	KT383812	KT383796	–	–	–	Ertz et al. (2015)
*Didymocyrtis ramalinae*	CBS 128019	KP170647	JQ238616	–	–	–	Trakunyingcharoen et al. (2014)
*Dlhawksworthia clematidicola*	MFLUCC 14-0910 T	NR_164267	NG_069556	NG_063681	MG829202	–	Wanasinghe et al. (2018)
*Edenia gomezpompae*	CBS 124106 T	NR_156217	NG_059202	–	–	–	Macías-Rubalcava et al. (2008)
*Edenia gomezpompae*	LVPEI 3225	KU578033	–	–	–	–	Unpublished
*Elongaticollum hedychii*	MFLUCC 18-1638	MT321796	MT321810	MT321803	MT328753	–	Tennakoon et al. (2020)
*Elongaticollum hedychii*	MFLUCC 17-2653 T	MT321797	MT321811	MT321804	MT328754	–	Tennakoon et al. (2020)
*Elongaticollum hedychii*	NCYUCC 19-0286	MT321798	MT321812	MT321805	MT328755	–	Tennakoon et al. (2020)
*Embarria clematidis*	MFLUCC 14-0976	MG828871	MG828987	MG829099	MG829194	–	Wanasinghe et al. (2018)
*Equiseticola fusispora*	MFLUCC 14-0522 T	NR_154010	NG_059249	NG_061238	MG520895	–	Abd-Elsalam et al. (2016)
*Galiicola baoshanensis*	HKAS 102234 T	NR_164300	NG_068613	NG_067698	MK359066	–	Phookamsak et al. (2019)
*Hydeomyces desertipleosporoides*	SQUCC 15259 T	NR_164295	NG_068287	NG_067692	MK290848	–	Maharachchikumbura et al. (2019)
*Italica achilleae*	MFLUCC 14-0959 T	NR_157518	NG_059862	NG_063683	MG829204	MG829255	Wanasinghe et al. (2018)
*Jeremyomyces labinae*	CBS 144617 T	NR_163362	NG_070463	–	–	MK442665	Crous et al. (2019)
*Juncaceicola luzulae*	MFLUCC 13-0780 T	KX449529	KX449530	KX449531	MG520898	–	Tennakoon et al. (2016)
*Kwanghwana miscanthi*	FU31017 T	MK503817	MK503823	MK503829	MT009126	–	Karunarathna et al. (2020)
*Leptospora clematidis*	MFLUCC 17-2148 T	NR_170811	MT214577	MT226690	MT394636	MT394696	Phukhamsakda et al. (2020)
*Longispora clematidis*	MFLU 15–1277 T	MT310625	MT214580	MT226693	MT394639	–	Phukhamsakda et al. (2020)
*Mauginiella scaettae*	CBS 239.58	MH857770	MH869303	–	–	–	Vu et al. (2019)
*Megacoelomyces sanchezii*	CMHUB 24030	MN429018	MN429020	MN429016	–	MN546863	Dos Santos et al. (2021)
*Melnikia anthoxanthi*	MFLUCC 14-1011 T	–	KU848204	KU848205	–	–	Wijayawardene et al. (2020)
*Murichromolaenicola chiangraiensis*	MFLUCC 17-1488 T	MN994582	MN994559	MN994605	MN998163	–	Mapook et al. (2020)
*Muriphaeosphaeria galatellae*	MFLUCC 14-0614 T	NR_154189	NG_059600	NG_061216	MG520900	–	Phukhamsakda et al. (2015)
*Neoophiobolus chromolaenae*	MFLUCC 17-1467 T	NR_168851	NG_068693	NG_070144	MN998166	–	Mapook et al. (2020)
*Neosetophoma camporesii*	MFLUCC 15-0682 T	KU302779	KU302778	NG_070320	MN654114	–	Wanasinghe et al. (2018)
*Neosphaerellopsis thailandica*	CPC 21659 T	NR_137954	NG_067289	–	–	–	Trakunyingcharoen et al. (2014)
*Neostagonospora arrhenatheri*	MFLUCC 15-0464 T	KX926417	KX910091	KX950402	MG520901	–	Thambugala et al. (2017)
*Neostagonosporella bambusicola*	KUMCC 20-0031 T	OP454331	OP454335	OP454058	–	–	Dissanayake et al. (2022)
*Neostagonosporella sichuanensis*	MFLUCC 18-1228 T	MH368073	MH368079	MH368088	MK313851	–	Yang et al. (2019)
*Neostagonosporella sichuanensis*	MFLU 18-1223	MH394690	MH394687	MK296469	MK313854	–	Yang et al. (2019)
*Neosulcatispora agaves*	CPC 26407 T	NR_154233	NG_070394	–	–	–	Crous et al. (2015)
***Neosulcatispora agavicola***	**KUNCC25-21586** T	**PZ835569**	**PZ835559**	**PZ835564**	**PZ830944**	**PZ830949**	**This study**
***Neosulcatispora agavicola***	**KUNCC25-21587**	**PZ835570**	**PZ835560**	**PZ835565**	**PZ830945**	**PZ830950**	**This study**
***Neosulcatispora agavicola***	**KUNCC25-21588**	**PZ835571**	**PZ835561**	**PZ835566**	**PZ830946**	**PZ830951**	**This study**
***Neosulcatispora hongheensis***	**KUNCC25-21589** T	**PZ835572**	**PZ835562**	**PZ835567**	**PZ830947**	**PZ830952**	**This study**
***Neosulcatispora hongheensis***	**KUNCC25-21590**	**PZ835573**	**PZ835563**	**PZ835568**	**PZ830948**	**PZ830953**	**This study**
*Neosulcatispora strelitziae*	CPC 25657 T	KX228253	KX228305	–	–	–	Crous et al. (2016)
*Nodulosphaeria aconiti*	MFLUCC 13-0728 T	NR_154236	KU708844	KU708840	KU708852	KU708856	Mapook et al. (2016)
*Ophiobolopsis italica*	MFLUCC 17-1791 T	NR_156678	NG_059856	NG_061281	MG520903	–	Phookamsak et al. (2017)
*Ophiobolus disseminans*	MFLUCC 17-1787	MG520941	MG520961	MG520980	MG520906	–	Phookamsak et al. (2017)
*Ophiosimulans tanaceti*	MFLUCC 14-0525	KU738890	KU738891	KU738892	MG520910	–	Tibpromma et al. (2016)
*Ophiosphaerella taiwanica*	NTUCC 17-024 T	NR_171846	MN082419	–	MN199124	–	Ariyawansa and Jones (2019)
*Paraleptospora chromolaenae*	MFLUCC 17-1481 T	NR_168852	NG_068694	NG_070145	MN998167	–	Mapook et al. (2020)
*Paraloratospora camporesii*	MFLU 18-0915 T	NR_170014	NG_073784	MN756635	MN756633	–	Hyde et al. (2020)
*Paraloratospora marina*	MFLUCC 19 0691 T	OQ130046	OQ130110	OQ130107	OQ357219	OQ162221	Devadatha et al. (2023)
*Paraophiobolus arundinis*	MFLUCC 17-1789 T	NR_156680	NG_069539	NG_061282	MG520912	–	Phookamsak et al. (2017)
*Paraphoma radicina*	CBS 111.79 T	NR_156556	NG_070446	EU754092	–	KF252180	Quaedvlieg et al. (2013)
*Parastagonospora nodorum*	CBS 259.49 T	KF251185	KF251688	–	GU456285	KF252192	Quaedvlieg et al. (2013)
*Parastagonosporella fallopiae*	CBS 135981 T	NR_160062	NG_067337	–	–	MH460547	Bakhshi et al. (2018)
*Phaeosphaeria thysanolaenicola*	MFLUCC 10-0563 T	NR_155642	NG_069236	KM434286	KM434295	KM434303	Phookamsak et al. (2014a)
*Phaeosphaeriopsis triseptata*	MFLUCC 13-0271	KJ522475	KJ522479	KJ522484	MG520919	KJ522485	Thambugala et al. (2014)
*Piniphoma wesendahlina*	CBS 145032 T	NR_163375	NG_070464	–	–	MK442676	Crous et al. (2019)
*Populocrescentia ammophilae*	MFLUCC 17-0665 T	NR_157535	NG_059875	NG_063690	MG829231	–	Wanasinghe et al. (2018)
*Populocrescentia rosae*	TASM 6125 T	–	MG829060	NG_062440	MG829232	–	Wanasinghe et al. (2018)
*Pseudoophiosphaerella huishuiensis*	MFLUCC 17-1453	MN994590	MN994567	MN994613	MN998171	–	Mapook et al. (2020)
*Pseudophaeosphaeria rubi*	MFLUCC 14-0259 T	NR_154351	NG_067543	NG_061255	MG520934	–	Hyde et al. (2016)
*Pseudostaurosphaeria chromolaenae*	MFLUCC 17-1490 T	NR_168854	NG_068696	NG_070147	MN998174	–	Mapook et al. (2020)
*Quixadomyces hongheensis*	KUMCC 20-0215 T	NR_172441	MW264194	NG_074964	MW256816	MW269529	Wanasinghe et al. (2021)
*Quixadomyces hongheensis*	HKAS112346	MW541826	MW541822	MW541833	MW556134	MW556136	Wanasinghe et al. (2021)
*Sclerostagonospora lathyri*	MFLUCC 14-0958 T	NR_158956	NG_069566	NG_063692	MG829235	–	Wanasinghe et al. (2018)
*Scolicosporium minkeviciusii*	MFLUCC 12-0089	–	KF366382	KF366383	–	–	Wijayawardene et al. (2013)
*Septoriella ammophilicola*	MFLU 17-2571 T	MN047087	NG_070468	NG_068403	MN077065	–	Dayarathne et al. (2020)
*Septoriella asparagicola*	MFLUCC 16-0379 T	NR_165908	NG_070081	NG_067708	MK443385	MK443387	Hyde et al. (2019)
*Septoriella garethjonesii*	MFLUCC 15-0469	KX926425	KX954390	KY205717	MG520923	KX898363	Thambugala et al. (2017)
*Septoriella hubertusii*	CBS 338.86 T	NR_155786	KF251733	–	–	KF252235	Quaedvlieg et al. (2013)
*Septoriella neoarundinis*	MFLUCC 15-0046	KY706140	KY706130	KY706135	KY706144	KY706147	Thambugala et al. (2017)
*Septoriella neodactylidis*	MFLUCC 14-0966 T	NR_157511	NG_069554	NG_061288	MG829199	MG829253	Wanasinghe et al. (2018)
*Septoriella paradactylidis*	MFLU 17-0498 T	NR_164251	NG_070411	–	–	–	Hyde et al. (2017)
*Septoriella verrucispora*	MFLUCC 19-0163 T	MK522508	MK522498	MK522504	MK523388	–	Zhang et al. (2019)
*Setomelanomma holmii*	CBS 110217	KT389542	GU301871	GU296196	GU349028	GU371800	Schoch et al. (2009)
*Setophoma caverna*	R150	MK511944	MK511965	–	–	–	Liu et al. (2019)
*Setophoma sacchari*	CBS 333.39 T	NR_145173	NG_057837	NG_062779	–	KF252250	de Gruyter et al. (2010)
*Setophoma sacchari*	MFLUCC 12-0241	KJ476145	KJ476147	KJ476149	KJ461318	–	Phookamsak et al. (2014b)
*Stagonospora foliicola*	CBS 110111	KF251256	KF251759	EU754118	–	–	Quaedvlieg et al. (2013)
*Sulcispora supratumida*	MFLUCC 14-0995 T	NR_160325	NG_067530	NG_065627	MH665366	–	Senanayake et al. (2018)
*Tintelnotia destructans*	CBS 127737 T	NR_147684	NG_058274	NG_063077	–	KY090683	Ahmed et al. (2017)
*Vittaliana mangrovei*	NFCCI 4251 T	NR_165870	NG_067809	NG_067664	MG767314	MG767315	Devadatha et al. (2019)
*Vrystaatia aloeicola*	CBS 135107	KF251278	KF251781	–	–	KF252283	Quaedvlieg et al. (2013)
*Wingfieldomyces cyperi*	CBS 141450 T	NR_155805	NG_059684	–	–	MK540101	Marin-Felix et al. (2019)
*Wojnowiciella kunmingensis*	KUMCC 18-0159 T	NR_164446	NG_070079	NG_067701	MK359071	MK359078	Phookamsak et al. (2019)
*Xenophaeosphaeria grewiae*	CBS 138867 T	NR_137944	NG_058124	–	–	–	Crous et al. (2014)
*Xenophoma puncteliae*	CBS 128022	JQ238617	JQ238619	–	–	–	Lawrey et al. (2012)
*Xenoseptoria neosaccardoi*	CBS 128665	KF251281	KF251784	–	–	KF252286	Quaedvlieg et al. (2013)
*Yunnanensis chromolaenae*	MFLUCC 17-1487 T	NR_168856	NG_068698	NG_070149	MN998178	–	Mapook et al. (2020)

Sequences obtained in this study are shown in bold. Type specimen or ex-type strain marked with ‘T’; unavailable data or sequences are marked with ‘–’.

### Sequence alignment and phylogenetic analyses

Verified sequences were initially used for BLASTn analyses, following which closely related sequences were downloaded from GenBank based on BLAST similarities (Table 1). Alignment of each locus was performed using MAFFT v. 7 (Katoh et al. 2019), using default conditions. It was then improved wherever necessary in the BioEdit v.7.0.5.2 software (Hall 1999). Phylogenetic analyses using both Maximum Likelihood (ML) and Bayesian Inference (BI) approaches were conducted via the CIPRES Science Gateway (Miller et al. 2010). For ML analyses, RAxML-HPC2 on XSEDE v.8.2.10 (Stamatakis 2014) was employed with default settings and 1,000 bootstrap replicates to assess branch support. Bayesian analyses were carried out using MrBayes on ACCESS (Ronquist et al. 2012), implementing the GTR+I+G substitution model. The MCMC algorithm was run for 2M generations, with trees sampled every 100 generations. The run terminated automatically once the average standard deviation of split frequencies fell below 0.01, and the first 25% of trees were discarded as burn-in. ML bootstrap values (MLBS) ≥ 70% and Bayesian posterior probabilities (BYPP) ≥ 0.95 are reported above the corresponding branches in the phylogenetic trees. To further evaluate the relationships among *Neosulcatispora agavicola*, *N. hongheensis* and the type species, *N. agaves*, an additional ML analysis was conducted using the ITS dataset in IQ-TREE. Branch support was assessed using 1,000 SH-aLRT replicates and 1,000 ultrafast bootstrap replicates. The resulting ITS-based phylogenetic tree is provided as Suppl. material 1. Final trees were visualized using FigTree v.1.4.0 (Rambaut and Drummond 2012) and annotated using either Microsoft PowerPoint (2007). GenBank accession numbers for all newly generated sequences are provided in Table 1.

## Results

### Phylogenetic analyses

In order to examine the evolutionary relationships of our new strains within *Phaeosphaeriaceae*, phylogenetic analyses were performed based on the combined SSU, LSU, ITS, *tef*1-α and *rpb*2 DNA sequences of 92 representatives of the genera in *Phaeosphaeriaceae* and two strains from *Quixadomyces hongheensis* in *Parapyrenochaetaceae* (HKAS112346, KUMCC 20-0215) as the outgroup taxon. The full dataset consisted of 4386 characters including gaps (SSU = 999 characters, LSU = 846 characters, ITS = 640 characters, *tef*1 = 873 characters, *rpb*2 = 1028 characters). The RAxML analysis of the combined dataset yielded a best scoring tree with a final ML optimization likelihood value of −39772.413931. The matrix had 1752 distinct alignment patterns, with 34.22% of undetermined characters or gaps. Parameters for the GTR + I + G model of the combined amplicons were as follows: Estimated base frequencies; A = 0.245050, C = 0.240226, G = 0.265660, T = 0.249064; substitution rates AC = 1.207364, AG = 3.503172, AT = 2.203036, CG = 0.825955, CT = 7.277140, GT = 1.000; proportion of invariable sites I = 0.618388; gamma distribution shape parameter α = 0.664524. The Bayesian analyses generated 20001 trees from which 15001 were sampled after 25% of the trees were discarded as burn-in. The alignment contained a total of 1754 unique site patterns.

**Figure 1.**
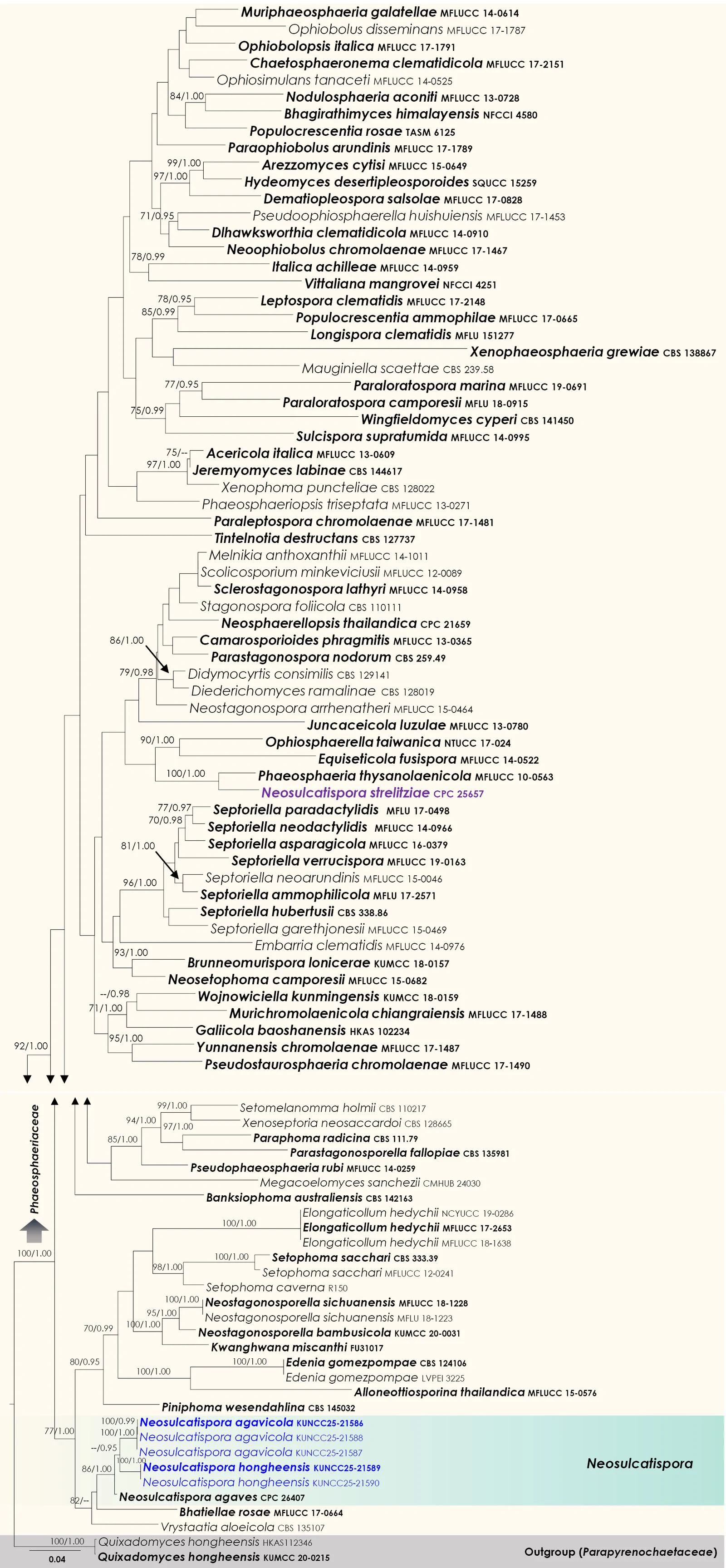
RAxML tree based on a combined dataset of partial SSU, LSU, ITS, *tef*1 and *rpb*2 DNA sequence analysis in *Phaeosphaeriaceae*. Bootstrap support values for ML (MLBS) equal to or greater than 70%, Bayesian posterior probabilities (BYPP) equal to or greater than 0.95 are shown as MLBS/BYPP above the nodes. The new isolates are in blue. Uncertain strains are in purple. The scale bar represents the expected number of nucleotide substitutions per site.

The ML and BI analyses produced congruent topologies for the focal lineages. Five newly obtained isolates were resolved within the clade containing the type species, *Neosulcatispora agaves* CPC 26407 (Fig. 1). Three isolates assigned to *N. agavicola* formed a lineage with 100% MLBS, and 0.99 BYPP statistical supports. Two isolates assigned to *N. hongheensis* also formed another fully supported monophyletic lineage (100% MLBS, and 1.00 BYPP). The two new species were resolved as separate clades, and their relationship received 0.95 BYPP, while ML support was below the reporting threshold. The clade comprising both new species and *N. agaves* received 86% MLBS and 1.00 BYPP.

The ex-type strain of *N. strelitziae* CPC 25657 was not recovered within the clade containing the type species of *Neosulcatispora*; instead, it was placed elsewhere in *Phaeosphaeriaceae* sister to *Phaeosphaeria thysanolaenicola* MFLUCC 10-0563 (Fig. 1). The present results support placement of the two new taxa in *Neosulcatispora* sensu stricto but raise questions regarding the current placement of *N. strelitziae*. No nomenclatural change is proposed for that species here because a dedicated analysis with broader sampling and complete locus coverage is required.

Only ITS and LSU sequences are currently available for the ex-type strain of *Neosulcatispora agaves* (CPC 26407); SSU and protein-coding sequences for *tef*1-α and *rpb*2 are unavailable. Therefore, direct comparisons between the two new species and *N. agaves* using protein-coding loci were not possible. In the ITS-based phylogeny, the three isolates of *N. agavicola* formed a distinct monophyletic lineage with 94.9% SH-aLRT and 100% ultrafast bootstrap support. The two isolates of *N. hongheensis* formed a monophyletic lineage with 99.2% SH-aLRT and 100% ultrafast bootstrap support. The two new species were resolved as distinct lineages and were separated from *N. agaves* (Suppl. material 1).

### Taxonomy

#### Neosulcatispora

Taxon classificationFungiPleosporalesPhaeosphaeriaceae

Crous & M.J. Wingf., Persoonia 35: 283 (2015)

B76AFA6B-C232-57BB-A966-E8D2E5D5B143

814930

##### Type species.

*Neosulcatispora agaves* Crous & M.J. Wingf., in Crous et al., Persoonia 35: 283 (2015).

##### Generic notes.

In the present five-locus phylogenetic analysis, the type species *Neosulcatispora agaves* forms a clade with *N. agavicola* and *N. hongheensis*. These species share pycnidial conidiomata with brown walls of *textura angularis*, phialidic conidiogenous cells and pigmented conidia. The placement of *N. strelitziae* outside this clade suggests that the genus, as previously applied, may not be monophyletic. *Neosulcatispora strelitziae* has erumpent, globose, dark brown to black with central ostiole conidiomata. Conidiophores reduced to conidiogenous cells, or with a supporting cell, lining the inner cavity, hyaline, smooth, ampulliform, phialidic, with periclinal thickening or tightly aggregated percurrent proliferations. solitary, golden-brown, smooth, ellipsoid, 0–1-septate conidia. The morphology and phylogeny of *N. strelitziae* should therefore be reassessed using additional loci and related genera before any recombination is proposed.

#### Neosulcatispora agavicola

Taxon classificationFungiPleosporalesPhaeosphaeriaceae

L.S. Dissan., Wanas., Phookamsak & J.C. Xu sp. nov.

B850CF85-9C04-5C8C-87A9-483CC8EC2FAF

865065

Fig. 2

##### Etymology.

The epithet refers to the host genus *Agave*, from which the fungus was isolated.

##### Holotype.

KUN-HKAS 155191.

##### Description.

***Saprobic*** on dry leaves of *Agave vera-cruz* (*Asparagaceae*). **Sexual morph**: Undetermined. **Asexual morph**: Coelomycetous. ***Conidiomata*** 250–300 × 180–220 μm (x̄ = 265.87 × 199.06 μm, n = 10), semi-immersed to erumpent through host epidermis, solitary, becoming aggregated, globose, glabrous, uni-loculate, with ostiolate, visible as dark brown dots on host surface. ***Ostiole*** central, minutely papillate, with pore-like opening, protruding through host surface. ***Conidiomata wall*** 30–50 μm (x̄ = 42.76 μm, n = 10) wide, comprising 3–4 layers of small, flattened, dark brown to black, pseudoparenchymatous cells, of *textura angularis* to *textura prismatica*, slightly paler towards the inner layer, outer layers composed of thick-walled, coriaceous, inner layers composed of flattened, thin-walled cells. ***Conidiophores*** reduced to conidiogenous cells; or with a supporting cell, lining the inner cavity, hyaline. ***Conidiogenous cells*** lining the inner cavity of the conidiomata, hyaline, smooth-walled, thin-walled, aseptate, ampulliform to lageniform or subcylindrical; some phialidic, with inconspicuous periclinal thickening, while others show tightly aggregated percurrent proliferations at the apex, producing conidia singly. ***Conidia*** 7–10.5 × 4–6 μm (x̄ = 7.97 × 4.89 μm, n = 50), solitary, varied in shape, ellipsoidal to subfusoid, or oblong, with obtuse or acute ends, straight to irregularly curved, initially hyaline, with two big polar guttules, becoming golden to brown, 1-euseptate, upper cell slightly shorter and wider than the lower cell, thick- and rough-walled, prominently striate, with striations covering the length of the conidium, becoming dark brown after discharge.

##### Culture characteristics.

Colony grows faster on PDA, reaching 20 mm in diameter after seven days at 25 °C under dark conditions, flat, spreading, with moderate aerial mycelium and margin irregular, producing brown-black pigment when mature. Sporulated after 30 days. ***In vitro*: *Stromatic*** 1240 × 1325 μm, immersed to semi-immersed on PDA, solitary or aggregated, with or without an ostiole, visible as dark brown to black dots. ***Conidiomata wall*** comprising brown cells of *textura angularis*. ***Conidiophores*** reduced to conidiogenous cells. ***Conidiogenous cells*** lining the inner cavity of the conidiomata, hyaline, smooth-walled, thin-walled, aseptate, ampulliform to lageniform or subcylindrical; some phialidic, with inconspicuous periclinal thickening, while others show tightly aggregated percurrent proliferations at the apex, producing conidia singly. ***Conidia*** 7.43–11.10 × 3.49–4.83 μm (x̄ = 9.02 × 4.16 μm, n = 40), solitary, subcylindrical, straight or slightly curved, initially hyaline, becoming 1-septate, brown, prominently striate, with striations covering the length of the conidium.

**Figure 2.**
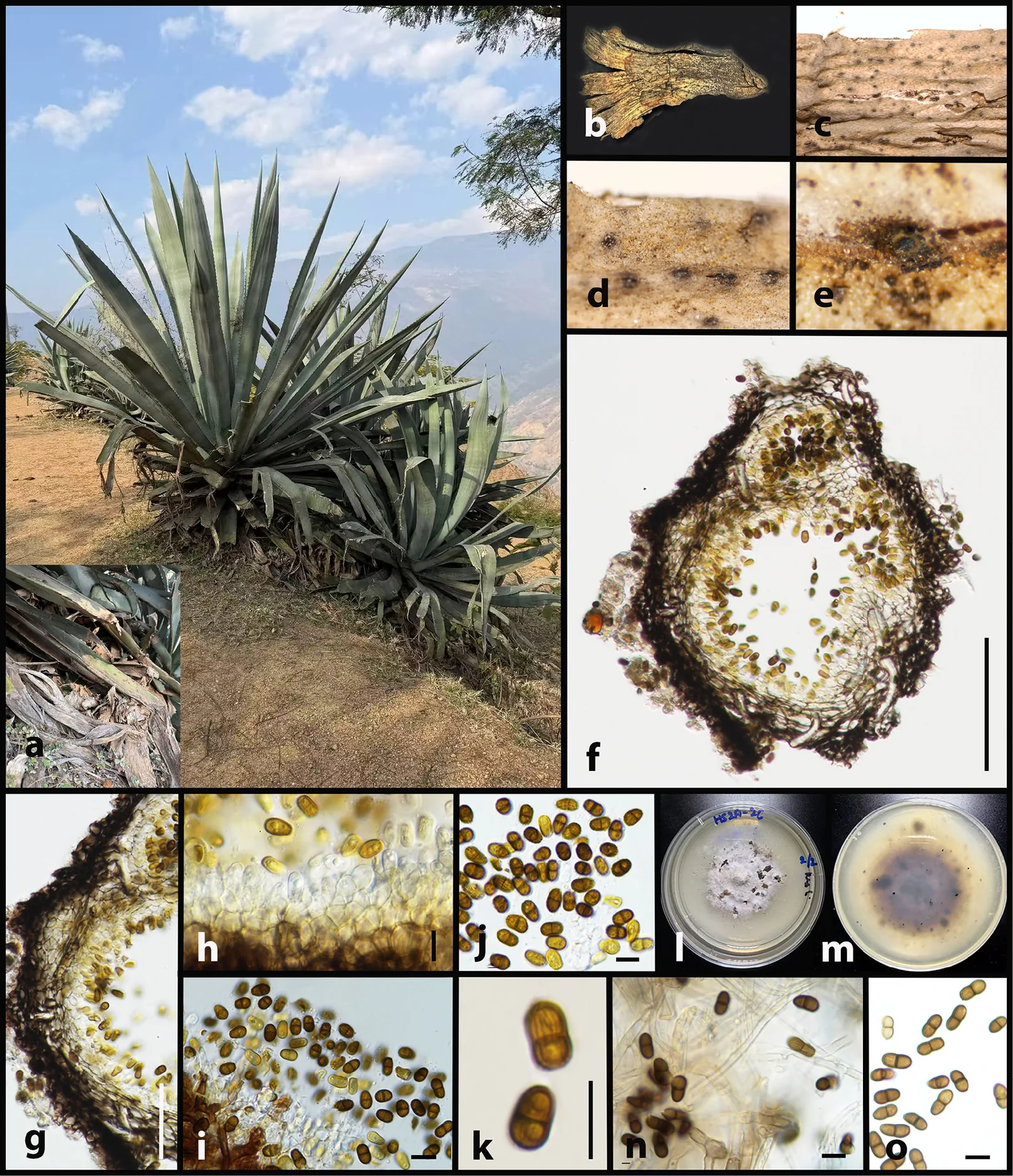
*Neosulcatispora agavicola* (holotype KUN-HKAS155191). **a**. *Agave vera-cruz* host and habitat; **b–e**. Black conidiomata on the host surface; **f**. Vertical section of a conidioma; **g**. Conidiomata wall; **h, i**. Conidiogenous cells and developing conidia; **j, k**. Conidia; **l, m**. Colonies on PDA from above (**l**) and below (**m**); **n, o**. Conidia on colonies. Scale bars: 100 μm (**f**); 50 μm (**g**); 10 μm (**h–k, n, o**).

##### Materials examined.

China • Yunnan, Honghe Hani and Yi Autonomous Prefecture, Honghe County, 23.2452°N, 102.1546°E, 762 m, on dry leaves of *Agave vera-cruz* (*Asparagaceae*), 14 January 2026, L.S. Dissanayake, HS2A1A (holotype, KUN-HKAS 155191), ex-type culture, KUNCC25-21586; • ibid., HS2A1B (isotype, KUN-HKAS 155192), ex-isotype culture, KUNCC25-21587, HS2A1C (KUN-HKAS 155193), living culture, KUNCC25-21588.

##### Note.

The three isolates representing *Neosulcatispora agavicola* form a fully supported, independent lineage (100% MLBS, 1.00 BYPP; Fig. 1) within *Neosulcatispora* sensu stricto. The species can be distinguished from *N. hongheensis* by its larger conidiomata and 1-septate mature conidia. It is morphologically similar to *N. agaves*, which produces prominently longitudinally striate, 1-septate conidia measuring (7–)9–11(–12) × (3.5–)4(–4.5) μm (Table 2). Because the conidial dimensions of the two species overlap substantially, the distinction of *N. agavicola* from *N. agaves* cannot be based on morphology alone. Although the conidiomata of *N. agavicola* are slightly smaller than the maximum diameter of approximately 300 μm reported for *N. agaves* (Table 2), this difference is insufficient by itself to justify species delimitation. *Neosulcatispora agavicola* and *N. agaves* are not recovered as immediate sister taxa in the combined phylogeny (Fig. 1). Pairwise nucleotide comparisons between *N. agavicola* and *N. agaves* revealed no differences in LSU (0/846 bp). Across 540 ITS positions, 12 nucleotide differences were detected when gap-containing positions were excluded (2.22%), whereas 23 positions differed when gaps were counted as differences (4.26%). Consistent with these differences, the ITS-based phylogeny recovered the three isolates of *N. agavicola* as a distinct monophyletic lineage with 94.9% SH-aLRT and 100% ultrafast bootstrap support, separate from *N. hongheensis* and *N. agaves* (Suppl. material 1). SSU, *tef*1-α and *rpb*2 sequences are unavailable for *N. agaves* preventing direct comparisons using these loci.

**Table 2.** Synopsis of accepted and proposed species assigned to *Neosulcatispora*.

**Species**	**Conidiomata (μm)**	**Conidia (μm)**	**Septation / ornamentation**	**Host and locality**	**References**
*N. agaves*	Up to ca. 300 μm diam.	(7–)9–11(–12) × (3.5–)4(–4.5)	1-septate; longitudinally striate	On leaves of *Agave vera-cruz* (*Asparagaceae*); La Réunion (an island in the Indian Ocean), France	Crous et al. (2015)
***N. agavicola***	**250–300 × 180–220**	**7–10.5 × 4–6**	**1-septate; longitudinally striate**	**On dry leaves of *Agave vera-cruz* (*Asparagaceae*); Honghe, Yunnan, China**	**This study**
***N. hongheensis***	**90–120 × 100–150**	**7.5–10 × 3.8–6**	**3–4-septate; longitudinally striate**	**On dry leaves of *Agave vera-cruz* (*Asparagaceae*); Honghe, Yunnan, China**	**This study**
*N. strelitziae*	Up to 350 μm diam.	(4–)5–7(–8) × (2.5–)3	0–1-septate; smooth	On leaves of *Strelitzia nicolai* (*Strelitziaceae*); Haga Haga, Eastern Cape, South Africa	Crous et al. (2016)

The new species are in bold.

In comparison with *Neosulcatispora hongheensis*, *N. agavicola* differs by 2/846 bp in LSU (0.24% difference), 20/540 bp in ITS (3.7% difference), 25/873 bp in *tef*1 (2.86% difference) and 46/891 bp in *rpb*2 (5.16% difference). These molecular differences, together with the clear differences in conidiomatal size and conidial septation, support the recognition of *N. agavicola* and *N. hongheensis* as distinct species.

#### Neosulcatispora hongheensis

Taxon classificationFungiPleosporalesPhaeosphaeriaceae

L.S. Dissan., Wanas., Phookamsak & J.C. Xu sp. nov.

12D60ED4-4E2A-5F3D-938F-918F1117EBCB

865066

Fig. 3

##### Etymology.

The epithet refers to Honghe Prefecture, Yunnan Province, China, where the fungus was collected.

##### Holotype.

KUN-HKAS155194.

##### Description.

***Saprobic*** on dry leaves of *Agave vera-cruz* (*Asparagaceae*). **Sexual morph**: Undetermined. **Asexual morph**: Coelomycetous. ***Conidiomata*** 90–120 × 100–150 μm (x̄ = 114.43 × 124.62 μm, n = 10), semi-immersed, to erumpent through host epidermis, solitary, scattered to clustered, becoming aggregated, globose to subglobose, or irregular in shape, visible as dark brown dots on host surface, uni- to bi-loculate, glabrous, with inconspicuous ostiole. ***Conidiomata wall*** 30–42 μm (x̄ = 37.14 μm, n = 10) wide, comprising 3–4 layers of broaden to flattened, brown to dark brown, pseudoparenchymatous cells, of *textura angularis*, slightly paler brown to hyaline towards the inner layer. ***Conidiophores*** reduced to conidiogenous cells; or with a supporting cell, lining the inner cavity, hyaline. ***Conidiogenous cells*** lining the inner cavity of the conidiomata, hyaline, smooth-walled, thin-walled, aseptate, ampulliform to lageniform or subcylindrical; some phialidic, with inconspicuous periclinal thickening, while others show tightly aggregated percurrent proliferations at the apex, producing conidia singly. ***Conidia*** 7.5–10 × 3.8–6 μm (x̄ = 8.68 × 4.76 μm, n = 50), solitary, oblong to subcylindrical, or ellipsoidal, straight to irregularly curved, initially hyaline, with two polar and smaller guttules, becoming 3–4-septate, golden to brown, thick- and rough-walled, prominently striate, becoming dark brown after discharge.

**Figure 3.**
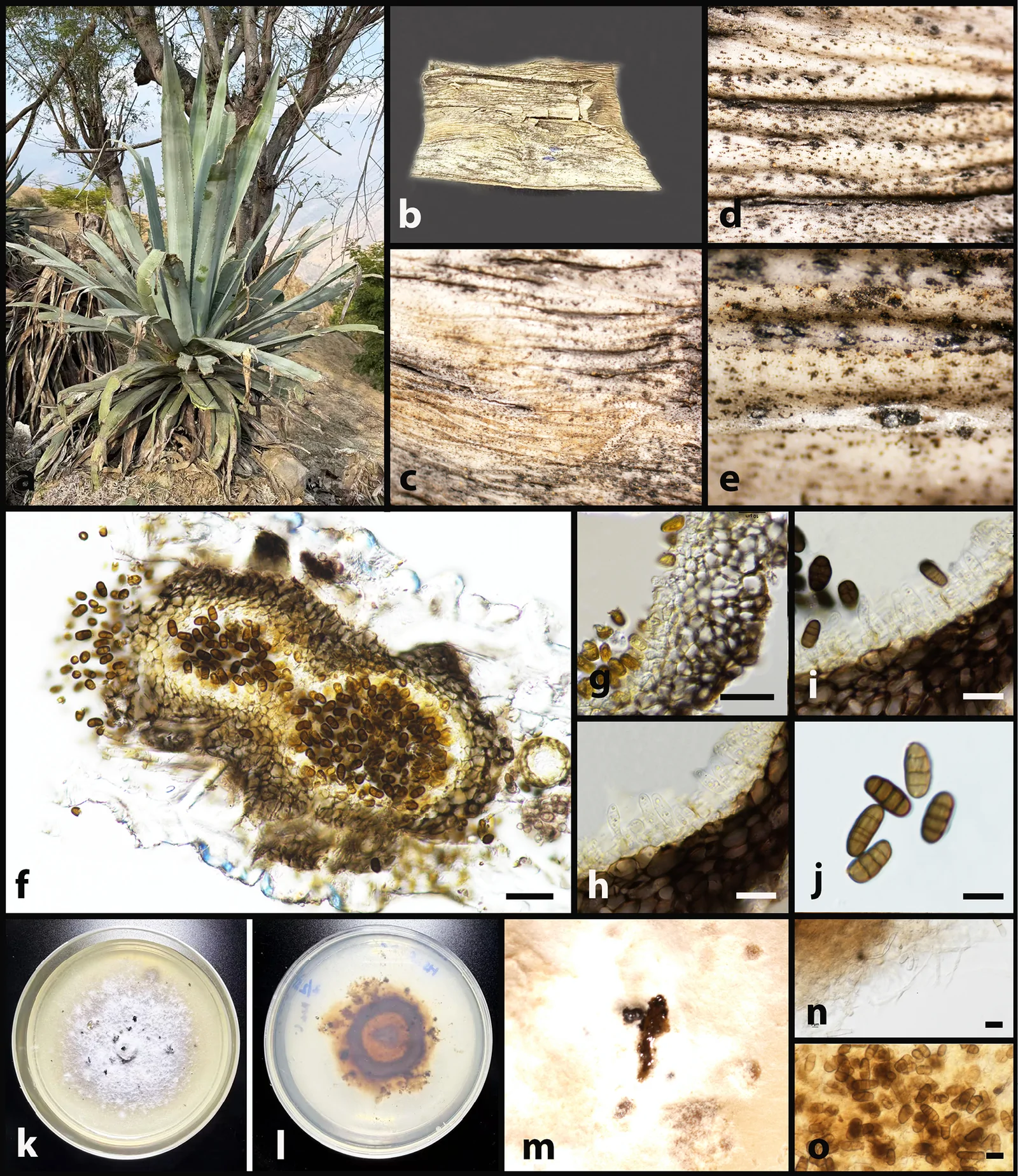
*Neosulcatispora hongheensis* (holotype KUN-HKAS 155194). **a**. *Agave vera-cruz* host and habitat; **b–e**. Black conidiomata on the host surface; **f**. Horizontal section of a conidioma; **g**. Conidiomata wall; **h, i**. Conidiogenous cells and developing conidia; **j**. Conidia; **k, l**. Colonies on PDA from above (**k**) and below (**l**); **m–o**. Conidioma and conidia on colonies. Scale bars: 50 μm (**f, g**), 10 μm (**h–j, n, o**).

##### Culture characteristics.

Colony grows faster on PDA, reaching 30 mm in diameter after seven days at 25 °C under dark conditions, flat, spreading, with moderate aerial mycelium and margin irregular, white surface and reverse golden brown, producing brown-black pigment when mature. Sporulated after 30 days. ***In vitro*: *Stromatic*** 1400 × 1700 μm, immersed to semi-immersed on PDA, solitary or aggregated, with or without an ostiole, visible as dark brown to black dots. ***Conidiomata wall*** comprising brown cells of *textura angularis*. ***Conidiophores*** reduced to conidiogenous cells. ***Conidiogenous cells*** lining the inner cavity of the conidiomata, hyaline, smooth-walled, thin-walled, aseptate, ampulliform to lageniform or subcylindrical; some phialidic, with inconspicuous periclinal thickening, while others show tightly aggregated percurrent proliferations at the apex, producing conidia singly. ***Conidia*** 10–15 × 4.2–7.4 μm (x̄ = 12.92 × 5.89 μm, n = 40), solitary, subcylindrical, straight or slightly curved, initially hyaline, becoming 3–4 septate, brown, prominently striate, with striations covering the length of the conidium.

##### Materials examined.

China • Yunnan, Honghe Hani and Yi Autonomous Prefecture, Honghe County, 23.2452°N, 102.1546°E, 762 m, on dry leaves of *Agave vera-cruz* (*Asparagaceae*), 14 January 2026, L.S. Dissanayake, HS2A2A (holotype, KUN-HKAS155194), ex-type culture, KUNCC25-21589; • ibid., HS2A2B (isotype, KUN-HKAS155195), ex-isotype culture, KUNCC25-21590.

##### Note.

The two isolates of *Neosulcatispora hongheensis* form a fully supported lineage (100% MLBS, and 1.00 BYPP; Fig. 1) within the clade containing *N. agavicola* and the type species, *N. agaves*. Morphologically, *N. hongheensis* is readily distinguished from these species by its smaller conidiomata and mature conidia with 3–4 septa, whereas *N. agaves* and *N. agavicola* produce mainly 1-septate mature conidia (Table 2). *Neosulcatispora strelitziae* has smooth, 0–1-septate conidia measuring (4–)5–7(–8) × (2.5–)3 μm (Table 2), which are narrower and less septate than those of *N. hongheensis*. However, *N. strelitziae* is phylogenetically distant from *N. hongheensis* and is recovered outside *Neosulcatispora* sensu stricto. Pairwise nucleotide differences between *N. hongheensis* and *N. agavicola* are provided in the notes under *N. agavicola*. The ITS-based phylogeny also recovered the two isolates of *Neosulcatispora hongheensis* as a well-supported monophyletic lineage with 99.2% SH-aLRT and 100% ultrafast bootstrap support, separate from *N. agavicola* and *N. agaves* (Suppl. material 1).

## Discussion

The present study introduces two new species, *Neosulcatispora agavicola* and *N. hongheensis*, within *Neosulcatispora* sensu stricto based on an integrative assessment of morphology and multi-locus phylogenetic evidence. Both species are represented by multiple isolates and form fully supported, independent lineages within the clade containing the type species, *N. agaves* (Fig. 1). Their placement in the genus is also supported by their coelomycetous asexual morphs, characterized by pycnidial conidiomata, brown conidiomatal walls composed of cells of *textura angularis*, phialidic conidiogenous cells and pigmented conidia (Crous et al. 2015).

Among the four species currently assigned to *Neosulcatispora*, *N. agaves*, *N. agavicola* and *N. hongheensis* share longitudinally striate conidia and have been recorded from leaves of *Agave vera-cruz*. In contrast, *N. strelitziae*, described from *Strelitzia nicolai* (Crous et al. 2016), has smooth conidia and is recovered outside the clade containing the type species in the present analysis. Longitudinal striation may therefore be a useful morphological character for recognizing *Neosulcatispora* sensu stricto. However, only three striate species and a small number of collections are currently known. Broader taxon and specimen sampling are required before this character can be regarded as phylogenetically conserved at the generic level.

Conidial septation is variable among the three species currently placed in *Neosulcatispora* sensu stricto. *Neosulcatispora agaves* and *N. agavicola* have predominantly 1-septate mature conidia, whereas *N. hongheensis* produces 3–4-septate conidia. Septation may therefore be useful for distinguishing species, provided that fully mature conidia are compared because septa may develop progressively during conidial maturation. The occurrence of all three striate species on *Agave vera-cruz* is noteworthy. *Neosulcatispora agaves* was reported from La Réunion, whereas *N. agavicola* and *N. hongheensis* were collected in southwestern China. Nevertheless, these few records are insufficient to demonstrate host specificity and may partly reflect limited collecting or host-sampling bias. These fungi should therefore not yet be regarded as *Agave*-associated specialists. Additional sampling of *Agave*, related plant hosts and other xerophytic vegetation is needed to determine whether this apparent host association is consistent.

Studies of other filamentous fungi have demonstrated that conidial surface properties can influence hydrophobicity, adhesion, aerial dispersal and interactions with substrates (Beever and Dempsey 1978; Stringer et al. 1991; Dague et al. 2008; Zhang et al. 2011; Whitehead et al. 2021). Comparative analyses have also suggested that fungal spore ornamentation may be associated with substrate-specific deposition and attachment (Calhim et al. 2018). However, these findings cannot presently be extrapolated to the longitudinal striation of *Neosulcatispora*. No ultrastructural, chemical, wettability or adhesion data are currently available for these species, and no adaptive or ecological function can therefore be assigned to the striation. Scanning electron microscopy, contact-angle measurements, adhesion assays and comparative desiccation experiments would be required to test whether the striation has a functional role or is primarily a taxonomically informative morphological character.

The phylogenetic positions of *Neosulcatispora agaves* and *N. strelitziae* should be interpreted carefully because both species are represented only by ITS and LSU sequences; SSU, *tef*1-α and *rpb*2 data are unavailable. In the concatenated analysis, their placements are therefore determined solely by the available ribosomal loci, whereas the five newly obtained isolates are represented by all five loci. The missing loci reduce the information available for estimating the placement and stability of these previously described species and prevent direct comparisons based on protein-coding genes. Consequently, the present topology provides a working hypothesis for the circumscription of *Neosulcatispora* sensu stricto rather than a definitive resolution of the relationships among all described species. Additional protein-coding sequences from *N. agaves* and *N. strelitziae* are required to test these relationships more robustly. A further taxonomically important result is the placement of the ex-type strain of *Neosulcatispora strelitziae* outside the clade containing the type species, *N. agaves*. The original description reported relatively low ITS similarity between *N. strelitziae* and *N. agaves*, despite their high LSU similarity (Crous et al. 2016). *Neosulcatispora strelitziae* differs morphologically from the species accommodated in *Neosulcatispora* sensu stricto by its smaller, smooth, 0–1-septate conidia. The present topology therefore suggests that *N. strelitziae* may not be congeneric with *N. agaves*. However, a formal taxonomic reassignment would be premature because the available sequence data are limited. Broader taxon sampling around the lineage containing the ex-type strain, together with additional protein-coding loci, will be required to determine its appropriate generic placement. Until such data become available, *Neosulcatispora* sensu stricto should be regarded as the lineage containing the type species, *N. agaves*, together with *N. agavicola* and *N. hongheensis*.

The discovery of these species also highlights the importance of the Honghe dry-hot valleys as an understudied niche of undescribed fungal diversity. Previous studies have documented numerous taxonomic novelties from diverse hosts and substrates in Yunnan Province (Dissanayake et al. 2024; Gao et al. 2024; Li et al. 2024; Luo et al. 2024; Phookamsak et al. 2024; Jiang et al. 2026; Sun et al. 2026), while a targeted survey of dead woody litter in the Honghe dry-hot valleys revealed substantial previously undocumented microfungal diversity (Wanasinghe et al. 2025). The present study extends these findings to xerophytic monocotyledonous hosts and demonstrates the value of habitat-focused fungal inventories. Continued surveys integrating well-documented voucher specimens, publicly accessible living cultures, detailed morphological observations, and multi-locus sequence data will be essential for resolving fungal diversity and evolutionary relationships in these ecologically distinctive environments.

## Supplementary Material

XML Treatment for Neosulcatispora

XML Treatment for Neosulcatispora agavicola

XML Treatment for Neosulcatispora hongheensis

## Additional information

### Artificial Intelligence (AI) use

The authors accept full responsibility for the content of the manuscript, including the disclosure of any use of AI.

No AI tools were used in the preparation of this manuscript.

### Author ORCIDs

L.S. Dissanayake https://orcid.org/0000-0003-2933-3127

R. Phookamsak https://orcid.org/0000-0002-6321-8416

S.S.N. Maharachchikumbura https://orcid.org/0000-0001-9127-0783

A.E. Al-Sabri https://orcid.org/0000-0001-8692-5730

J. Xu https://orcid.org/0000-0002-2485-2254

D.N. Wanasinghe https://orcid.org/0000-0003-1759-3933

### Data availability

The datasets generated for this study can be found in the NCBI, GenBank and MycoBank. All of the relevant accessible information is available in the main text.

## Supplementary materials

Supplementary material 1 ITS-based phylogenetic tree Data type pdf https://binary.pensoft.net/file/1821570This dataset is made available under the Open Database License (http://opendatacommons.org/licenses/odbl/1.0/). The Open Database License (ODbL) is a license agreement intended to allow users to freely share, modify, and use this Dataset while maintaining this same freedom for others, provided that the original source and author(s) are credited. Lakmali S. Dissanayake, Rungtiwa Phookamsak, Sajeewa S. N. Maharachchikumbura, Ahmed E. Al-Sabri, Jianchu Xu, Dhanushka N. Wanasinghe
