# Supplementary material for: Taxonomic novelties of two Neosulcatispora species (Pleosporales, Phaeosphaeriaceae) associated with Agave vera-cruz plants in a dry-hot valley of Yunnan, China
Source: MycoKeys. 2026 Oct 26;142:157–84. doi: 10.3897/mycokeys.142.213343 (PMC13728823; doi:10.3897/mycokeys.142.213343)
Supplement: Supplementary file 1 [file mycokeys-142-157-s001.pdf]

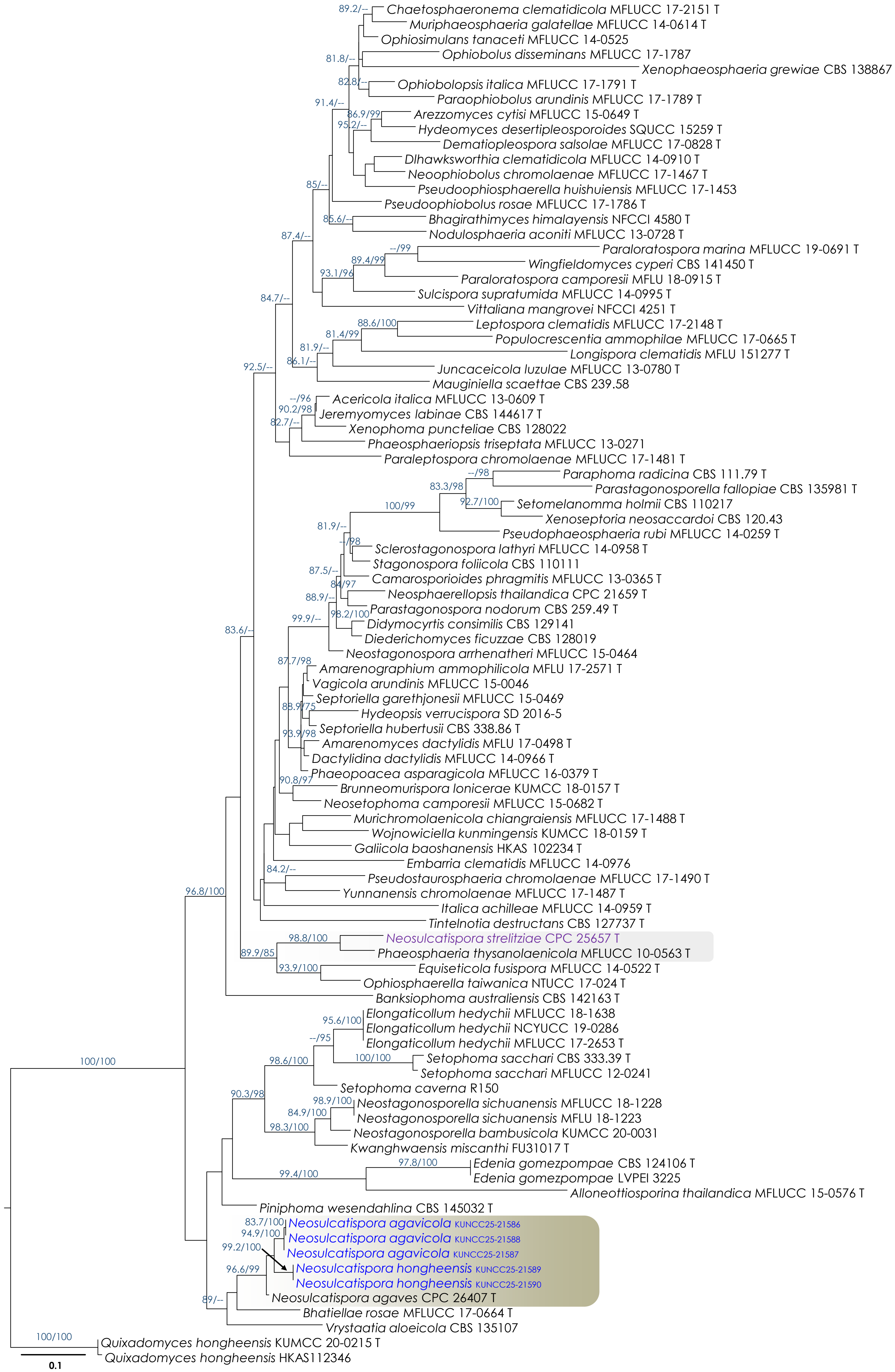

89.2/--
Chaetosphaeronema clematidicola MFLUCC 17-2151 T
Muriphaeosphaeria galatellae MFLUCC 14-0614 T
Ophiosimulans tanaceti MFLUCC 14-0525
81.8/--
Ophiobolus disseminans MFLUCC 17-1787
Xenophaeosphaeria grewiae CBS 138867 T
82.8/--
Ophiobolopsis italica MFLUCC 17-1791 T
Paraophiobolus arundinis MFLUCC 17-1789 T
91.4/--
86.9/99
Arezzomyces cytisi MFLUCC 15-0649 T
95.2/--
Hydeomyces desertipleosporoides SQUCC 15259 T
Dematiopleospora salsolae MFLUCC 17-0828 T
Dlhawksworthia clematidicola MFLUCC 14-0910 T
Neoophiobolus chromolaenae MFLUCC 17-1467 T
85/--
Pseudoophiosphaerella huishuiensis MFLUCC 17-1453
Pseudoophiobolus rosae MFLUCC 17-1786 T
85.6/--
Bhagirathimyces himalayensis NFCCI 4580 T
87.4/--
Nodulosphaeria aconiti MFLUCC 13-0728 T
--/99
Paraloratospora marina MFLUCC 19-0691 T
89.4/99
Wingfieldomyces cyperi CBS 141450 T
93.1/96
Paraloratospora camporesii MFLU 18-0915 T
Sulcispora supratumida MFLUCC 14-0995 T
84.7/--
Vittaliana mangrovei NFCCI 4251 T
88.6/100
Leptospora clematidis MFLUCC 17-2148 T
81.4/99
Populocrescentia ammophilae MFLUCC 17-0665 T
81.9/--
Longispora clematidis MFLU 151277 T
92.5/--
86.1/--
Juncaceicola luzulae MFLUCC 13-0780 T
Mauginiella scaettae CBS 239.58
--/96
Acericola italica MFLUCC 13-0609 T
90.2/98
Jeremyomyces labinae CBS 144617 T
82.7/--
Xenophoma puncteliae CBS 128022
Phaeosphaeriopsis triseptata MFLUCC 13-0271
Paraleptospora chromolaenae MFLUCC 17-1481 T
--/98
Paraphoma radicina CBS 111.79 T
83.3/98
Parastagonosporella fallopiae CBS 135981 T
100/99
92.7/100
Setomelanomma holmii CBS 110217
Xenoseptoria neosaccardoi CBS 120.43
81.9/--
Pseudophaeosphaeria rubi MFLUCC 14-0259 T
--/98
Sclerostagonospora lathyri MFLUCC 14-0958 T
Stagonospora foliicola CBS 110111
87.5/--
Camarosporioides phragmitis MFLUCC 13-0365 T
84/97
Neosphaerellopsis thailandica CPC 21659 T
88.9/--
Parastagonospora nodorum CBS 259.49 T
99.9/--
98.2/100
Didymocyrtis consimilis CBS 129141
83.6/--
Diederichomyces ficuzzae CBS 128019
Neostagonospora arrhenatheri MFLUCC 15-0464
87.7/98
Amarenographium ammophilicola MFLU 17-2571 T
Vagicola arundinis MFLUCC 15-0046
Septoriella garethjonesii MFLUCC 15-0469
88.9/75
Hydeopsis verrucispora SD 2016-5
Septoriella hubertusii CBS 338.86 T
93.9/98
Amarenomyces dactylidis MFLU 17-0498 T
Dactylidina dactylidis MFLUCC 14-0966 T
Phaeopoacea asparagicola MFLUCC 16-0379 T
90.8/97
Brunneomurispora lonicerae KUMCC 18-0157 T
Neosetophoma camporesii MFLUCC 15-0682 T
Murichromolaenicola chiangraiensis MFLUCC 17-1488 T
Wojnowiciella kunmingensis KUMCC 18-0159 T
Galiicola baoshanensis HKAS 102234 T
Embarria clematidis MFLUCC 14-0976
84.2/--
Pseudostaurosphaeria chromolaenae MFLUCC 17-1490 T
96.8/100
Yunnanensis chromolaenae MFLUCC 17-1487 T
Italica achilleae MFLUCC 14-0959 T
Tintelnotia destructans CBS 127737 T
98.8/100
Neosulcatispora strelitziae CPC 25657 T
89.9/85
Phaeosphaeria thysanolaenicola MFLUCC 10-0563 T
93.9/100
Equiseticola fusispora MFLUCC 14-0522 T
Ophiosphaerella taiwanica NTUCC 17-024 T
Banksiophoma australiensis CBS 142163 T
95.6/100
Elongaticollum hedychii MFLUCC 18-1638
Elongaticollum hedychii NCYUCC 19-0286
--/95
Elongaticollum hedychii MFLUCC 17-2653 T
100/100
100/100
Setophoma sacchari CBS 333.39 T
98.6/100
Setophoma sacchari MFLUCC 12-0241
Setophoma caverna R150
90.3/98
98.9/100
Neostagonosporella sichuanensis MFLUCC 18-1228
84.9/100
Neostagonosporella sichuanensis MFLU 18-1223
98.3/100
Neostagonosporella bambusicola KUMCC 20-0031
Kwanghwaensis miscanthi FU31017 T
97.8/100
Edenia gomezpompae CBS 124106 T
99.4/100
Edenia gomezpompae LVPEI 3225
Alloneottiosporina thailandica MFLUCC 15-0576 T
Piniphoma wesendahlina CBS 145032 T
83.7/100
Neosulcatispora agavicola KUNCC25-21586
94.9/100
Neosulcatispora agavicola KUNCC25-21588
99.2/100
Neosulcatispora agavicola KUNCC25-21587
96.6/99
Neosulcatispora hongheensis KUNCC25-21589
Neosulcatispora hongheensis KUNCC25-21590
89/--
Neosulcatispora agaves CPC 26407 T
Bhatiellae rosae MFLUCC 17-0664 T
Vrystaatia aloeicola CBS 135107
100/100
Quixadomyces hongheensis KUMCC 20-0215 T
Quixadomyces hongheensis HKAS112346
0.1
